# Nursing Professional Self-Concept: A Scoping Review Protocol

**DOI:** 10.3390/nursrep13010005

**Published:** 2023-01-04

**Authors:** Inês Franco Almeida, Rafael Alves Bernardes, Liliana Baptista Sousa, Paulo Santos-Costa, Rosa Silva, Joana Bernardo, Elaine Santana, Amorim Rosa

**Affiliations:** 1 The Health Sciences Research Unit: Nursing (UICISA: E), Nursing School of Coimbra (ESEnfC), 3004-011 Coimbra, Portugal; 2 Portugal Centre for Evidence-Based Practice: A JBI Centre of Excellence (PCEBP/JBI), 3000 Coimbra, Portugal

**Keywords:** nursing, professional self-concept, nursing students, nurses, scoping review

## Abstract

Nurses are considered one of the largest professional groups in healthcare, generating positive health outcomes for people at various stages of life. A significant impact on the construction of the professional self—or professional self-concept—is considered to exist through the educational process, influenced by factors such as the family and societal expectations often presented by teachers, tutors, and peers. Improving professional self-concept in nursing can offer specific gains in personal, relational, social, and interpersonal communication skills, favoring evolution in the academic and clinical path. This scoping review aims to map the literature related to the state of knowledge regarding professional self-concept in nursing. This scoping review will follow JBI recommendations with the PCC mnemonic and report its findings through PRISMA-ScR using a specific instrument made by the researchers. Providing healthcare complying with high scientific standards requires the professional to have enough self-confidence in his work and skills. The explicit acknowledgement of professional self-concept is essential for any educational tutor or experienced mentor to promote mental health and academic and professional performance.

## 1. Introduction

Across people’s lifetimes, nurses are essential in promoting positive healthcare outcomes and delivering effective interventions and are also considered one of the largest professional groups [1]. Their global preparedness for clinical challenges is substantially important and is increased by contemporary challenges, namely by older patients with multiple chronic comorbidities [2]. Particularly for nursing students, due to the complexity and nature of the nursing practice, increased responsibility throughout the years is more pronounced. A significant impact on the construction of the professional self—or professional self-concept—is considered to exist throughout the educational process, influenced by factors such as the expectations of family and society [3], often represented by advisors, teachers, tutors, and peers [4]. One of the main goals of nursing education and professional enhancement is the improvement of professional self-concept [5].

Moreover, we can assert that their professional self-concept may influence nursing students’ and nurses’ mental health and well-being in the same way as other relevant job-related factors such as job satisfaction, burnout, and stress [6]. This construct has been studied and approached by the multidimensional self-concept theory by Cowin [6], among others, which has also developed an assessment tool to measure it. For some authors, professional self-concept is defined as an individual’s perception of self as a professional person, which affects different aspects of professional performance [7].

A positive professional self-concept is considered a protective factor against depression and anxiety [8]. A good and high professional self-concept is considered a protective and effective factor for the occurrence of burnout in the nursing profession, and the higher the professional self-concept, the lower the burnout [9]. A high professional self-concept is also related to increased self-esteem and self-efficacy, creating a sense of well-being and accomplishment [10], which provides a healthier mental health context for coping with daily life and professional challenges.

Improving professional self-concept in nursing presents specific gains in personal, relational, interpersonal communication, and social skills [11] favoring academic and clinical path evolution. Nurses with high professional self-concept are more accountable for their patients [12] and work outcomes and see patients more respectfully and interestedly [13]. It is also worth mentioning that high professional self-concept positively influences the acceptance and demand for evidence-based practice in nursing [14].

Adverse effects of poor professional self-concept lead nurses to develop reduced clinical skills, show lower job satisfaction [15], and have a higher intention to rotate between services and institutions [16]. This turnover and institutional liability lead to high daily costs for the health system, care providers, and patients [17]. Additionally, this fact may lead to consequences such as early dropout from the profession [18] and decreased academic quality and performance [19].

According to a study conducted in China, nursing students in the country showed a high professional self-concept [20]; such results may be the result of cultural differences such as language, beliefs, values, and educational and economic factors.

A study performed in Iran highlighted that the workplace environment influences nurses’ professional self-concept; this means that an oppressed work environment and bullying present a negative impact on professional self-concept, as verified in Iran [21].

In order to further underline the importance and timeliness of this topic, the professional self-concept of nurses who had received care regarding the COVID-19 pandemic was assessed and was found to be lower than that of their professional colleagues who had not received the same care. This difference was more pronounced in the knowledge and care dimensions, which may be related to the lack of preparation and absence of concrete standards to deal with a pandemic on a global scale. It may also be related to working under high pressure, as well as the lack of qualified human resources to provide care and not having enough time to update their knowledge [22].

This concept has recently received some attention as a structural theoretical construct among many professional sectors [23]. Despite this, through a preliminary search in MEDLINE, the Cochrane Database of Systematic Reviews, and JBI Evidence Synthesis, it was possible to verify that this topic has been almost unstudied, and there is no current or review currently underway about it. We also researched PROSPERO, and zero results were obtained in the survey. We noticed a limited number and disparity of tools available to assess this construct, as well as a limited number of translations, validations, and adaptations to other cultures and countries and a restricted range of interventions to improve the professional self-concept. Due to the importance of this theme for training students and future professionals, it is considered important to map and organize the knowledge studied on this topic, taking into account the different international realities of nurses and their self-concepts. For a proper understanding of the professional self-concept, it is essential to map, characterize, and analyze its many definitions and understandings in the literature.

Thus, this Scoping Review aims to map the literature related to the definition of professional self-concept in nursing, how is it measured, and what interventions are performed to improve it.

Review Question(s):

The review questions are “How is professional self-concept defined in nursing?”, “How is professional self-concept measured?”, and “What types of interventions are available to improve professional self-concept in students and nurses?”.

## 2. Materials and Methods

The proposed scoping review protocol will follow the updated JBI methodology for scoping reviews [24].

Findings will be reported according to the Preferred Reporting Items for Systematic Reviews and Meta-Analyses (PRISMA) guidelines, utilizing the extension for scoping reviews (PRISMA-ScR) [25].

### 2.1. Eligibility Criteria

Following the recommendations of JBI for scoping reviews, the criteria consider the PCC mnemonic for scoping reviews, where P stands for ”participants”, C for ”concept”, and C for ”context” [24].

We have selected the following inclusion criteria: concerning the participants, this review will consider studies that include undergraduate nursing students and nurses in any field of action and specialty; regarding the concept, this review will consist of studies that explore the development and concept of the professional self-concept in nursing; concerning the context, this review will consider articles, independent of the country of the study, conducted in any setting such as clinical, research and tutoring, among others; regarding the types of sources, this scoping review will consider all study typologies, i.e., quantitative, qualitative, and mixed methods. In this approach, both primary studies and reviews (published and unpublished) will be mapped.

### 2.2. Search Strategy

In order to develop the search strategy, two reviewers will consider the aim and the review questions with the help of an experienced third reviewer, who will also assist in resolving potential conflict scenarios.

An initial limited search of CINAHL completed via EBSCOhost was undertaken to identify publications on the topic. In order to develop a search strategy, we used the text words constrained in the titles and abstracts of relevant articles, as well as the index terms used to describe the articles (Table 1). Each database’s search strategy will be adapted, including all identified keywords and index terms. The reference list of all included sources of evidence will also be screened for additional studies.

The studies’ languages will be limited to English, Portuguese, and Spanish to guarantee a quality selection process and quality data extraction, minimizing interpretation errors.

The databases to be searched will include MEDLINE (via PubMed), CINAHL complete (via EBSCOhost), Academic search complete (Via EBSCOhost), Mediclatina (via EBSCOhost), Psychology and Behaviour Sciences Collection (via EBSCOhost), Cochrane Central Register of Controlled Trials (Via Cochrane Library), Cochrane Database of Systematic Reviews (Cochrane Library), SciELO, and Scopus. The search for unpublished studies, namely grey literature, will include OpenGrey and RCAAP.

Quantitative studies will include experimental studies (randomized controlled trials, non-randomized controlled trials, and quasi-experimental studies) and observational studies (with descriptive, exploratory, and analytical designs). Further quantitative studies included will be dissertations and theses, reports, government publications, and documents from organizations.

### 2.3. Study Selection

Following the search, all identified citations will be collected and uploaded into Mendeley V1.19.8 (Mendeley Ltd., Elsevier, Amsterdam, The Netherlands), and duplicates will be removed. After that, the citations will be imported into Rayyan QCRI (Qatar Computing Research Institute (Data Analytics), Doha, Qatar) for screening by two independent reviewers, who will assess the titles and abstracts according to the inclusion criteria. Afterwards, the potentially eligible studies will be retrieved, and the full texts will be evaluated in detail according to the inclusion criteria by two independent reviewers. Any disagreements that arise between the reviewers at each stage of the selection process will be resolved through discussion or with a third reviewer.

### 2.4. Data Extraction

Findings will be extracted from a specific instrument developed by the research team (Table 2). The draft data extraction tool will be modified and revised as necessary while extracting data from each included evidence source.

Study authors will be contacted for further information about missing data.

### 2.5. Data Analysis and Presentation

Data will be presented in tables, as seen in the previous point. Data extracted from the different studies will include the title, author, year of publication, country of origin, type of study, and objectives. The data extracted regarding the studies will consist of the professional self-concept definition, theoretical framework used, population, and the implications of professional nursing self-concept.

The scoping review is expected to be finalized in the month of July 2023, considering that the process of database searching is currently ongoing.

## 3. Discussion

In this protocol, we have described the process to develop a scoping review to map the definition, measurement tools, and interventions related to professional self-concept. Nevertheless, we acknowledge some possible limitations of this strategy, namely the fact that only English, Portuguese, and Spanish-language studies will be included. In order to avoid limiting ourselves to program types that are specific to one culture or have changed over time, we will not exclude papers based on countries or publication dates.

## 4. Conclusions

We expect that this review will allow a detailed analysis and clarification of the meaning of professional self-concept in nursing and map the respective impact in practice. We also hope that it will promote future research, highlighting its importance and the need to act and deepen this concept in both nursing students and nurses so that, in this way, we will better prepare nurses, the foundation of our health services.

## Figures and Tables

**Table 1:** Search strategy for EBSCOhost (CINAHL complete). The search was conducted on 13 September 2022.

Search	Query	Record Retrieved
#1	(AB (“professional self-concept”) OR (“professional self concept”) OR (“professional selfconcept”)) OR (TI (“professional self-concept”) OR (“professional self concept”) OR (“professional selfconcept”))	110
#2	(TI (Nurs *)) OR (AB (Nurs *))	613,645
#3	(TI((MM “Students, Nursing”) OR (“nursing students”) OR (“student nurses”) OR (“undergraduate student nurses”) OR (“pre-registration nursing student”) OR (“nurse students”) OR (“nurse student”) OR (“nursing student”))) OR (AB((MM “Students, Nursing”) OR (“nursing students”) OR (“student nurses”) OR (“undergraduate student nurses”) OR (“pre-registration nursing student”) OR (“nurse students”) OR (“nurse student”) OR (“nursing student”)))	39,687
#4	#1 AND #2 AND #3	34
Filters Applied	Language: English, Portuguese, Spanish	25

# = Research line, * = Truncation. Note: A similar strategy will be used for the remaining databases.

**Table 2:** Data extraction tool.

Evidence Source Details and Characteristics
Author(s)
Year of publication
Type of Study
Population and sample size
Study setting
Study aims/goals
Definition and/or characterization of professional self-concept
professional self-concept-related interventions reported:
How was professional self-concept measured?

## Data Availability

Not applicable.
